# Innovative Therapeutic Strategies for Cystic Fibrosis: Moving Forward to CRISPR Technique

**DOI:** 10.3389/fphar.2018.00396

**Published:** 2018-04-20

**Authors:** Michele Marangi, Giuseppa Pistritto

**Affiliations:** ^1^Department of Economic Strategy of Pharmaceutical Products, Italian Medicines Agency, Rome, Italy; ^2^Department of Systems Medicine, University of Rome Tor Vergata, Rome, Italy

**Keywords:** cystic fibrosis, CTFR mutation, ivacaftor, lumacaftor, gene therapy, CRISPR gene editing

## Abstract

One of the most revolutionary technologies in recent years in the field of molecular biology is CRISPR-Cas9. CRISPR technology is a promising tool for gene editing that provides researchers the opportunity to easily alter DNA sequences and modify gene function. Its many potential applications include correcting genetic defects, treating and preventing the spread of diseases. Cystic fibrosis (CF) is one of the most common lethal genetic diseases caused by mutations in the CF transmembrane conductance regulator (CFTR) gene. Although CF is an old acquaintance, there is still no effective/resolutive cure. Life expectancy has improved thanks to the combination of various treatments, but it is generally below average. Recently, a significant number of additional key medications have become licensed in Europe for the CF treatment including CFTR modulators. But innovative genomically-guided therapies have begun for CF and it is predictable that this will lead to rapid improvements in CF clinical disease and survival in the next decades. In this way, CRISPR-Cas9 approach may represent a valid tool to repair the CFTR mutation and hopeful results were obtained in tissue and animal models of CF disease.

## Introduction

Mutations within the gene encoding for the chloride ion channel CFTR results in cystic fibrosis (CF), the most common autosomal recessive genetic disease in the Caucasian population. CFTR gene encodes the CFTR protein, an adenosine triphosphate binding chloride anion channel located in the apical membrane of exocrine epithelial cells which allows the regulation of secretion of chloride and bicarbonate (O’Sullivan and Freedman, 2009). About 2000 mutations have been identified in the CFTR gene since its discovery in Kerem et al. (1989). CF has an incidence of 1 in 2500 live births with a predominance in those of northern European descent (Welsh et al., 2001).

Cystic fibrosis disease presents several symptoms since may affect several organs (Ashlock and Olson, 2011). Into this matter, bronchi and lungs are affected by recurrent bronchitis and bronchopneumonia, triggered by particular bacteria such as *Pseudomonas aeruginosa* and *Staphylococcus aureus*. The permanence of these bacteria causes infection and chronic inflammation of the lungs, with their progressive deterioration and a gradual decline in respiratory function, up to respiratory insufficiency (critical impairment of the intake of oxygen and elimination of carbon dioxide).

## Genetics

The gene CFTR responsible for CF is located at 7q31.2, ‘F508del’ (deletion of three bases coding for phenylalanine at the 508^th^ position) is the first most common mutation (Fanen et al., 2014). Traditional six classes of mutations are cataloged collecting each mutation with similar characteristics of altered production and/or function of the CFTR protein (**Table 1**). The variety of different mutations in the CFTR gene can be divided on the basis of their effects on the CFTR protein into class I (defective production due to premature protein truncation) (e.g., G542X), class II (defective processing/trafficking) (e.g., F508del), class III (defective regulation/gating) (e.g., G551D), class IV (defective conductance) (e.g., R117H), class V (reduced synthesis) (e.g., A455E), and class VI (reduced stability of protein at cell surface) (e.g., c.120del23) mutations (Elborn, 2016). The first three (I, II, III) are considered to be severe because they determine a slight or no adjustment of the ionic transposition while the other three (IV, V, VI) are mild because are associated with residual function of the protein and therefore to a milder form of disease.

**Table 1:** Classification of CTFR mutations, their impact on the protein.

Mutation class	Class 1	Class 2	Class 3	Class 4	Class 5	Class 6
Common mutations	G542X	F508del	G551D	R117H	A455E	4326deITC
	Gly542X	N1303K	G551S	R334W	2789 + 5G-A	N287Y
Frequency (%)	10%	70%	4%	3%	3%	
Disfunction	No stable RNA	CTFR trafficking defects	Defective CTFR regulation	Decreased CFTR conductance	Reduced CTFR synthesis	Decreased CFTR stability
**CFTR protein**						
Membrane location	No	Very low	Present	Present	Reduced	Reduced
Outcome	No	Poor	Poor	Partly	Good	Good
Therapeutic proposals	Read-through agents	Correctors+Potentiators	Potentiators	Correctors Potentiators	Stabilizers Amplifiers	Stabilizers Stabilizers
Licensed drugs		Lumacaftor/ivacaftor F508del	Ivacaftor	Ivacaftor R117H		
**Clinical conditions**	More severe diseases			Less severe diseases		

*Clinical features associated with the mutations and corrective therapeutic approaches*.

## Therapy

Although survival of CF patients is greatly improved, the evolution of pulmonary disease is still the main cause of morbidity and mortality in this disease. Several therapies including mucolytics, inhaled antimicrobials, systemic anti-inflammatories, and nutritional support are the mainstays of CF treatment, and these supportive therapies are largely responsible for the marked improvement in life expectancy over time. In particular, the corticosteroids (such as dexamethasone and prednisone) and non-steroidal anti-inflammatory drugs (such as ibuprophen) may prevent the morbidity and the progressive lung deterioration in CF.

However, anti-inflammatory therapies currently used in CF are addressed to the correction of events downstream of the basic defect of the disease (Belcher and Vij, 2010). In recent years, numerous *in vitro* and *in vivo* studies have been focused to the functional deficit of the CFTR protein and the underlying CFTR anion channel defect.

The ivacaftor drug (Kalydeco commercial name) was the first orally bioavailable CFTR potentiator, approved by the Food and drug Administration (FDA) for the G551D mutation (class III) (Kapoor et al., 2014). Later, Kalydeco was approved for eight other mutations including G1244E, G1349D, G178R, G551S, S1251N, S1255P, S549N, and S549R (Martiniano et al., 2016). The most recent is the R117H, for which the ivacaftor can also be administered to children of at least 2 years of age (Davies et al., 2016).

Ivacaftor is active only on rare CFTR mutations. For example, among the nearly 2,000 mutations of the CFTR gene, G551D affects only 4% of the world’s patients and R117H 3%, while 70% coexists with the delta-F508 mutation. Recently, the FDA has approved a new drug, always labeled Vertex: Orkambi, a mix between ivacaftor and lumacaftor, a CFTR protein corrector that has been tested in a clinical trial on 1,108 patients over the age of 12 years homozygous F508del. Lumacaftor is able to interact directly with the CFTR protein reducing the mis-folding of the defective F508del mutant CFTR protein and allowing the proper trafficking of CFTR to the cell surface. This activity can then be amplified by the ivacaftor that prolongs the open state of the F508del CFTR, thus increasing channel opening time. Based on the individual mechanisms, the combination of lumacaftor and ivacaftor was proposed to correct both, including protein trafficking as well as channel gating abnormalities (Bulloch et al., 2017). Orkambi effectiveness was evaluated in two clinical studies which showed a reduction of 39% in the number of exacerbations (flares-up) requiring hospital admission or antibiotic therapy of CF patients when compared with placebo. However, some Orkambi clinical outcomes were not considered statistically significant (only a slight increase in the respiratory function and failure of sweating test) (Davies et al., 2016). The therapeutic approaches through correctors/potentiators of CFTR protein need to get better compounds. Other studies are in progress for medicines amplifying the intracellular CFTR protein, stabilizing CFTR at the cell membrane or bypassing the CFTR protein by inhibiting the epithelial sodium channel ENaC or stimulating other chloride channels (Fajac and De Boeckc, 2017).

## Beyond Ivacaftor and Lumacaftor, New Hopes from Gene Therapy

Cystic fibrosis is a monogenic and autosomal recessive disease and this precondition has motivated the development of gene therapy-based strategies (Griesenbach et al., 2004). CF gene therapy consists in delivering of DNA or RNA nucleic acids encoding the CFTR protein or repairing of the CFTR gene (genome editing) or the CFTR mRNA (mRNA editing). Gene transfer into the lung is difficult due to extracellular barriers (mucus, mucociliar clearance, immune responses) and intracellular barriers (nuclear membrane). Since cloning of the CFTR gene 27 clinical trials involving about 600 patients were completed (Alton et al., 2016).

In gene strategy a functional copy of the gene is transported into the cells in order to program them to begin making the functional copies. While in gene therapy a correct version of the CFTR DNA sequence is delivered to the nucleus, in mRNA therapy a correct version of the CFTR DNA sequence is delivered to the cytoplasm. Although a normal protein is made in both cases, the approaches based on mRNA delivery have the potential advantage of not needing to overcome the nuclear membrane barrier (Alton et al., 2016).

The greatest obstacle to clinical success is the efficacy of gene delivery (Carlon et al., 2017). There are many approaches to improve this aspect, but viral and non-viral vectors are the most used vectors to carry nucleic acids into the cells.

Viral vectors are more efficient, because they infect the cells and are able to overcome at least some of the barriers. Virus is a potentially perfect vector for transporting genetic material because it is able to evade the immune system, attack cells, reprogram them in order to replicate virus genome. In gene therapy, the virus is modified to make it safe. Adenoviruses and adeno-associated viruses (AAVs) have a natural tropism for the lungs but the induced immune response to the viral vector reduces efficacy and duration of expression (Alton et al., 2016). Recent studies have demonstrated that it is possible to expand AAV tropism, reduce immunogenicity and enhance CFTR expression levels and persistence in the lung (Hart and Harrison, 2017). Lentiviral vectors were also utilized as vectors, but there are uncertainties about their safety profile and immunogenicity on repeat dosing. Furthermore, lentiviral vectors have no natural lung tropism and, therefore, require pseudotyping with appropriate envelope proteins to facilitate lung gene transfer (Alton et al., 2016). The most commonly protein used for this purpose is the vesicular stomatitis virus G (VSV-G) protein but it has safety concerns when used into clinical trials. For this reason, other proteins were studied such as the baculoviral protein GP64, proteins from Ebola or Marburg filoviruses, the HA protein from influenza virus and the F and HN protein from Sendai virus (Alton et al., 2016).

Conversely, non-viral vectors, such as liposomes or nanoparticles, are safe and allow repeated administration, but there is a need to improve nucleic acid delivery (Hart and Harrison, 2017). Recently, the UK CF Gene Therapy Consortium (GTC) has completed a double-blinded, placebo-controlled multi-dose phase IIb trial with 12 years or older age patients with moderate or mild lung disease. This trial has demonstrated that gene delivery was well-tolerated but benefit of liposome delivery was modest (Carlon et al., 2017).

## Repairing the CFTR Mutation: Gene Correction with Genome-Editing Nucleases

The possibility to cure the basic defect at the root, substituting the defective gene represents the molecular basis of the so-called “gene editing.” Recent advent in this technique has enabled a new more efficient tool compared with gene therapy by directly correcting the specific genetic lesions underlying disease. Whereas in gene therapy a new functional gene is transferred into the cells to replace a defective gene, gene editing works by repairing the defective gene at the DNA level.

Thanks to this technique it has become possible to intervene on the DNA at a level of precision before impossible, acting with real molecular scissors to cut the helix of the DNA at the desired point and replace a stroke (White et al., 2017). The repair of a defective gene at its original locus has two major advantages. First of all, the corrected gene remains under control of its endogenous promoter, therefore guaranteeing life-long expression and natural regulation in the cell. Moreover, depending on the delivery vehicle used, gene correction has the potential to avoid the involvement of foreign DNA, thus reducing the risk of insertional mutagenesis. Gene editing uses engineered nucleases, or “molecular scissors.” They are ZFN (Zinc Finger Nucleases), TALEN (Transcription Activator-Like Effector Nuclease) and the type II bacterial CRISPR/Cas9 (Clustered Regularly Inter-spaced Short Palindromic Repeats/CRISPR-Associated). Among them, CRISPR/Cas9 is considered the best efficient gene-editing technology as the efficiencies of ZFN and TALEN are low and time-consuming (Cong et al., 2013; Wang et al., 2013).

There are two components to the CRISPR system: a molecule known as a “guide RNA” (gRNA), suitably designed, which has the same sequence as the target site in the genome, and a “nuclease” (a DNA-cutting molecule) called Cas9. CRISPR is the name attributed to DNA segments containing short repeated sequences (clustered regularly interspaced short palindromic repeats), discovered within prokaryotic cells (Jusiak et al., 2016). The function of CRISPR has been revealed by the discovery of the existence of a complex of genes associated with these sequences, called Cas (contraction of crisper-associated), that encode for protein putative nucleases abling to cut DNA. In this way, the CRISPR/Cas association constitutes a prokaryotic immune system that confers resistance to foreign genetic elements providing a sort of acquired immunity. This system can be used to create RNA-guided libraries capable of appearing virtually in any gene sequence. You build an RNA-guide by fusing a bacterial RNA (TracrRNA) and an RNA complementary to the sequence you want to modify (crRNA). This system can be simplified by fusing crRNA and tracrRNA sequences to produce a synthetic chimeric single-guided RNA (sgRNA). When expressed in the cell, the gRNA mates with Cas9 and drives it on the target sequence: Cas9 cuts the DNA in the desired site and activates the DNA repair processes for homologous recombination. If the correct sequence of the gene to be modified is introduced into the cell, the homologous recombination will take this sequence as a mold and will definitively correct the mutated gene. The gRNA will bind to the target genomic site through complementary base pairing and will help bring in Cas9 to the target site to make a cut to the DNA creating site-specific double-strand breaks (DSBs).

In a second step, the DSBs are repaired using the cell’s endogenous system. Cells repair DSBs using the non-homologous end joining (NHEJ) or homology-directed repair (HDR) pathways. The first mode acts quickly and in a very effective way by ligating the broken strands together without the need for a homologous template. However, this pathway presents high probability of generating insertions or deletions (indels) at the DSBs. If the rupture occurs within a gene that produces a protein, the process can prevent the protein from being properly transcribed and translated. Alternatively, the HDR pathway requires a homologous user-provided DNA sequence, either from a plasmid or from single-stranded oligonucleotides to serve as a donor template for repair of both broken strands in a high-fidelity manner.

**FIGURE 1:**
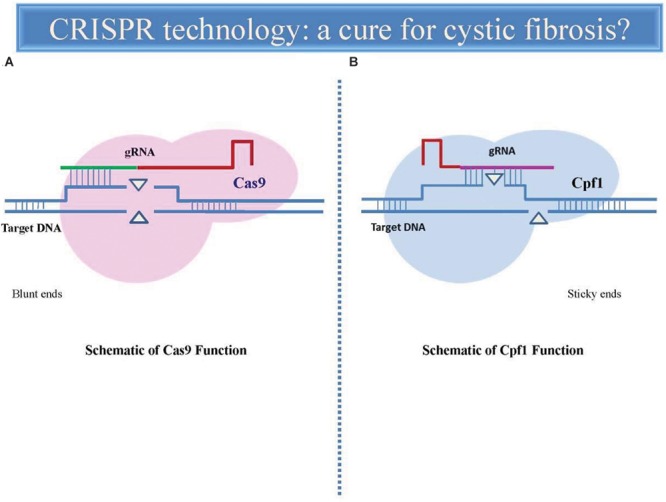
Comparative representation of CRISPR-Cas9 and CRISPR-Cpf1-mediated genome editing. The gRNA directs endonuclease Cas9 **(A)** to the target DNA sequence (blue) where it induces a double-strand break, leading to a sequence deletion. Cas9 uses a structural region of gRNA as a handle (red) and a variable targeting region (green) which identifies the target sequence to match and cleave. Cas9 can be specifically directed to the any target site of genome simply by modifying the sequence of the gRNA. Cpf1 endonuclease **(B)** contains a shorter and single identified nuclease domain (CRISPR-RNA), in contrast to the two nuclease domains present in Cas9. Cpf1-crRNA efficiently cleaves target DNA without the requirement for any additional RNA species. Cpf1 generates a staggered cut, in contrast to the blunt ends generated by Cas9. In both cases, the DSBs are subsequently repaired by two major cellular mechanisms, non-homologous end joining (NHEJ) and homology-directed repair (HDR).

A new tool in CRISPR genome editing, called Cpf1, has stimulated great interest for its attributes differing from Cas9: The Cpf1 gRNA is markedly shorter and requires only a single RNA molecule to cut DNA while Cas9 needs two RNA ones. This aspect makes easier the *in vitro* synthesis of gRNA and allow better its engineering into viral vectors. The proteins also cut DNA at different places, offering researchers more options when selecting an editing site. Cas9 cuts both strands in a DNA molecule at the same position, leaving behind ‘blunt’ ends. Cpf1 leaves one strand longer than the other, creating ‘sticky’ ends that are easier to work with (**Figure 1**). These sticky ends bring information that can target DNA insertion that is much more controllable and also improve the efficiency of CRISPR gene editing (Liu et al., 2017).

CRISPR/Cas9 was used to correct the genetic defect at the origin of Duchenne muscular dystrophy, caused by deletions of dystrophin gene, in a mouse model (Nelson et al., 2016) and in induced potential stem cells (iPSCs) derived from a patient lacking gene using CRISPR-Cas9 (Li H.L. et al., 2015). More recently, the new gene-editing CRISPR-Cpf1, has successfully corrected Duchenne muscular dystrophy in both a mouse model and DMD cell models derived from patients (Zhang et al., 2017).

Altering DNA in human embryos is also possible. Some scientist from China in 2015 published a reporting the alteration of DNA using abnormal human embryos previously rejected for *in vitro* fertilization use. They have limited success in correcting a mutation that causes the disease of beta thalassemia (mutation beta 41–42) using CRISPR/Cas9 (Liang et al., 2015). Some embryos have seen correcting the mutation in all the cells of which they were composed, while in other cases the so-called “mosaic embryos” appeared, where some cells were modified and others were not. Similar approach in human embryos was also used for the correction of heritable MYBPC3 mutation implicated in hypertrophic cardiomyopathy (Ma et al., 2017).

Promising applications of CRISPR/Cas9 technique were also performed on CF. Schwank et al. (2013) demonstrated the full restoration of CFTR protein functionality using the technique in a model of cultured intestinal stem cells (organoids) obtained from CF pediatric patients. These cultures lack the ability to respond to forskolin stimulation by swelling, mimicking important aspects of CF disease *in vitro*. The researchers corrected the mutation at the CFTR locus resulting in normal response to forskolin induced swelling. This application was an extension of similar research using mouse tissue, whereby the intestine products were successfully grafted into the mouse hosts (Yui et al., 2012).

Likewise, correction of CFTR mutation has been performed in iPSCs via CRISPR/Cas9 approach (Crane et al., 2015). iPSCs were obtained by reprogramming somatic skin fibroblasts obtained from CF patients into an embryonic stem cell state, transfected with a CFTR/Cas9 gRNA vector and subsequently differentiated toward a proximal airway epithelial cells Restoration of CFTR conductivity was shown similarly to wild-type iPSC derived lung epithelial cells. Furthermore, CFTR was able to change the conformation necessary for cell membrane translocation through N-glycosylation process (Firth et al., 2015). Stem cell niches have been identified in the lung, mostly of them give hospitality to the so-called bronchioalveolar stem cells. Therefore, in principle, it may be possible to obtain stem lung cells from a CF patient, engineering them with Crispr/Cas9 to correct the CFTR mutation, and reinsert them into one of those lung niches where stem cells find their suitable microenvironment for their survival and growth. Viral and non-viral delivery vehicles are employed for achieving Crispr/Cas9 expression into the airway epithelium cells. The most common used is the AAV vector (Kotterman and Schaffer, 2014). However, AAV delivery system seem inappropriate for this purpose, due to the discrete size of the CRISPR/Cas9 system which must be integrated into the target genome of the transfected cells; non-viral (lipidic or polymeric) vectors seem more suitable, also because they do not integrate into the host genome and therefore are free from the risk of possible transgene integration or induction of secondary tumors (Li L. et al., 2015). The ideal route of administration for introducing CRISPR/Cas9 into the lung is represented by aerosol delivery devices combined with nanoparticle suspensions, although the inhaled therapy risks to be entrapped and not overcome the formidable barrier represented by the dense and viscous pathological mucus that dominates the epithelium target (Ruge et al., 2013). In any case the elective route of administration must be local (by aerosol), since a systemic delivery based on intravenous administration, for anatomic reasons, reaches the alveolar area of the lung, which is devoted to gas exchange, and not the ciliated cells of the surface epithelium of the bronchial tree where CFTR protein is expressed.

## Conclusion

The introduction of new drugs such as ivacaftor and lumacaftor improved the quality of life and increased the life expectation of CF patients. Nevertheless, their use is not a decisive therapy. Gene therapy is able to restore working versions of the CFTR protein, but, although the simplicity of the gene therapy concept, the replacement with health gene presents technical barriers that are still unresolved. Despite being in its early stages, encouraging results have established the potential utility of CRISPR approach for CF therapy. Since CF is caused by a constellation of mutations, development of individualized autologous pulmonary models using patient specific cells could be necessary for a personalized therapy in patients. In this way, the treatment can be readily tailored to target an individual patient’s mutations. Seen in this light, CRISPR strategy may represent an important tool for CF treatment in the foreseeable future.

## Author Contributions

GP is researcher and Pharmacology teacher in Dept of “System Medicines,” University of Tor Vergata, Rome, and recently she has also joined AIFA as executive manager. She is an expert in Pharmacology, in particular in drugs involved in the control of the respiratory apparatus. In the review, she has described CRISPR gene editing, the new evolution of gene therapy, as a potential therapy for cystic fibrosis. Some preliminary CRISPR experimental approaches lead us to believe that this is a promising technique as a strategy for future disease treatment of cystic fibrosis. MM is an executive manager in Italian Medicines Agency (AIFA) and an expert in Pharmacology. He is involved in the new drug assessment for European centralized procedures (EMA) and he is mainly focused on the scanning for the new and emerging products/technology for healthcare. His contribution in the review was focused in particular on gene therapy and the process which has led to the discovery of new emerging therapy.

## Conflict of Interest Statement

The authors declare that the research was conducted in the absence of any commercial or financial relationships that could be construed as a potential conflict of interest. The reviewer PM and handling Editor declared their shared affiliation.
